# Ontogenetic changes in the long bone microstructure in the nine-banded armadillo (*Dasypus novemcinctus*)

**DOI:** 10.1371/journal.pone.0215655

**Published:** 2019-04-25

**Authors:** Christian Thomas Heck, David J. Varricchio, Timothy J. Gaudin, Holly N. Woodward, John R. Horner

**Affiliations:** 1 Department of Anatomy and Cell Biology, Oklahoma State University Center for Health Sciences, Tulsa, Oklahoma, United States of America; 2 Department of Earth Sciences, Montana State University, Bozeman, Montana, United States of America; 3 Department of Biology, Geology, and Environmental Science, University of Tennessee at Chattanooga, Chattanooga, Tennessee, United States of America; 4 Chapman University, Orange, California, United States of America; Perot Museum of Nature and Science, UNITED STATES

## Abstract

Analysis of ontogenetic changes in long bone microstructure aid in vertebrate life history reconstructions. Specifically, osteohistological examination of common fauna can be used to infer growth strategies of biologically uncommon, threatened, or extinct vertebrates. Although nine-banded armadillo biology has been studied extensively, work on growth history is limited. Here we describe long bone microstructure in tibiae and femora of a limited ontogenetic series of nine- banded armadillos (*Dasypus novemcinctus*) to elucidate patterns of bone growth. The cortex of the smallest individual is composed of compacted coarse cancellous bone (CCCB) and woven tissue. Extensive cortical drift is driven by periosteal erosion and further compaction of trabeculae resulting in an increase in the amount of CCCB. The cortex of the largest specimens is primarily CCCB with thickened endosteal bone and thin outer cortices of lamellar and parallel-fibered tissue. The outer cortices of the largest individuals are interpreted as an external fundamental system (EFS) indicating a cessation of appositional bone growth corresponding to skeletal maturity (i.e. asymptotic or adult size). The EFS forms in femora prior to tibiae, indicating femoral growth rates begin decreasing earlier than tibial in *D*. *novemcinctus*. Growth trends in common fauna like the nine-banded armadillo can be used as a foundation for understanding life histories of related, but uncommon or extinct, species of cingulates.

## Introduction

Recent molecular work suggests that Xenarthra is one of the four original clades of placental mammals [1–6]. Xenarthra is composed of sloths (Folivora), anteaters (Vermilingua), and armadillos (Cingulata). Cingulata contains all modern armadillos (Dasypodidae and Chlamyphoridae) and two extinct sub-families nested within Chlamyphoridae: Glyptodontinae and Pampatheriinae [7,8]. Currently, only eight of the 20 extant species of armadillo, including the nine-banded armadillo, are listed as Least Concern in the International Union for Conservation of Nature Red List of Threatened Species [9], and an additional five species lack sufficient data for assessment [10,11]. General biological, ecological, and physiological characteristics of a species must be understood in order to successfully implement conservation strategies [11,12]. Despite a recent increase in armadillo studies, gaps in armadillo research continue to inhibit conservation efforts due to difficulties in observing and tracking individuals in the wild and maintaining individuals in captivity [11,13–16]. Here, we describe growth patterns in wild *Dasypus novemcinctus* through analysis of long bone microstructure for their potential utility in future ecological, biological, and paleontological studies of cingulates.

Analysis of bone microstructure can elucidate life history details of animals that are difficult to observe in the wild because bone microstructure can reveal phylogenetic, biomechanical, ontogenetic, and environmental signals [17–27]. In addition, cyclical growth marks and annuli (e.g., lines of arrested growth or LAGs) within the bone indicate annual cessation of growth or, in the case of annuli, a slowing of appositional bone growth, and patterns of vascularity can be used to interpret relative growth rates by way of Amprino’s Rule [28–31]. Previous work on bone microstructure in armadillos focused primarily on armadillo dermal armor [32–37]. However, several studies include descriptions of bone microstructure in the appendicular skeleton of armadillos. Queckett [38] described osteonal arrangement in the humerus of *Dasypus peba* and the tibia and mandible from *Glyptodon clavipes*. Quekett [38] noted that the humerus consisted of obliquely oriented Haversian canals and the tibia of *G*. *clavipes* featured small osteons relative to the size of the tibia. Recently, Straehl *et al*. [39] examined phylogenetic differences in the long bone microstructure throughout Xenarthra. The extensive sample from Straehl *et al*. [39] encompassed various long bones from a broad range of taxa, including the nine-banded armadillo. However, comparing long bone microstructure across multiple species is difficult when ontogenetic studies are absent, especially because bones may undergo drastic morphological changes (e.g. muscle scarring, trochanteric growth, lengthening and/or thickening of shaft) through ontogeny. Cortical drift shapes the bone by resorption, differential deposition of new bone tissue, and perimedullary remodeling [40].

Nine-banded armadillos are a relatively new and yet common faunal element in the southern United States, emigrating northward from Central America into Texas in the mid nineteenth century [41–44]. Their range has continued to expand north through Tennessee, Nebraska, South Carolina, and individuals have been found as far north as South Dakota and Minnesota [42,45–49]. According to Humphrey [50], and as corroborated by Taulman and Robbins [49], annual precipitation may be more of a limiting factor than temperature. Changes in climate could extend the armadillo’s potential range further north or alter growth dynamics [14]. Understanding broad patterns of growth dynamics in the nine-banded armadillo can aid in elucidating geographical differences in growth and the effect of climate and environment on armadillo bone development. The study by Straehl *et al*. [39] histologically sampled the femur and humerus from two individuals of nine-banded armadillos and an additional humerus from a third individual. All three nine-banded armadillos examined in that study were categorized as adults by the researchers based on epiphyseal fusion and, after histological examination, the presence of an external fundamental system (EFS). Thus far, this is the only extensive histological analysis done on armadillos. Here we build upon that study by describing the hindlimb bone microstructure of a limited ontogenetic series of *D*. *novemcinctus* in order to build foundational work on armadillo bone growth dynamics.

## Materials and methods

### Institutional abbreviations

University of Tennessee-Chattanooga, Chattanooga, Tennessee, USA (UTCM); Sam Noble Museum of Natural History, Norman, Oklahoma, USA (OMNH).

### Materials and methods

Skeletal elements sampled for this study were obtained from the collections of University of Tennessee-Chattanooga and Sam Noble Museum of Natural History. No animals were sacrificed for this study, therefore, IACUC approval was not necessary. We sampled left femora and tibiae from six nine-banded armadillos (*Dasypus novemcinctus)* representing a limited ontogenetic series. Empirical intraskeletal studies have demonstrated that larger weight bearing bones preserve higher apposition rates than non-weight bearing bones and thus, can be used as a proxy for yearly increases in body mass [26, 51–54]. Individuals sampled were originally collected as road kill. Road kill specimens have been used in demographic and morphological nine-banded armadillo studies to alleviate difficulties in tracking and capturing specimens, although accurate body mass measurements are difficult to obtain due to the oft-scattered condition of these specimens [55]. Loughry and McDonough [14] also note that although roadkill specimens are convenient, they may not represent an accurate view of population dynamics. We assume bone microstructure is not influenced by sidedness; left and right elements of the same type in an individual share similar osteohistological characters [56]. Quantitative morphological details of sampled elements can be found in Table 1. Previously, epiphyseal fusion has been used to assess skeletal maturity based on observations of extant mammals e.g. [57]. However, Parfitt [58] suggests epiphyseal fusion is the result of growth cessation and not the cause. Therefore, a partially fused epiphysis would indicate the long bone is skeletally mature and growth has ceased. The epiphyses of UTCM 801, UTCM 802, and OMNH 39188 were unfused in both elements and we tentatively identify these individuals as juveniles. The femur of UTCM 801 is lacking a distal epiphysis, but all other elements are complete. Conversely, the long bone epiphyses of UTCM 1557 and OMNH 40173 are nearly fused but have faintly visible suture lines and the long bones of OMNH 40175 are completely fused. UTCM 1557, OMNH 40173, and OMNH 40175 are thus labeled as adults. Collection date, collection locality, length measurements, and body mass of specimens are summarized in Table 1. Complete cast replicas of each element were produced prior to histological preparation by molding and casting procedures outlined in Padian and Lamm [26]. Macro-photographs of individual elements were taken using a Nikon D60 DSLR equipped with an AF-S Nikkor 18- 55mm zoom lens. Additionally, specimens from OMNH were 3D scanned prior to sampling using a NextEngine 3D Scanner Ultra HD and compiled with NextEngine ScanStudio.

**Table 1:** Collection and morphological data for sampled specimens.

Museum	Specimen	Sex	Ontogenetic Stage*	Femur Length (mm)	Tibia/Fibula Length (mm)	Year Collected	Location Collected
UTCM	801	NA	Juvenile	84.10	67.03	1999	Candler, GA
	802	NA	Juvenile	77.20	61.67	1999	Candler, GA
	1557	M	Adult	95.59	78.23	2007	Franklin, TN
OMNH	39188	M	Juvenile	91.50	74.00	2010	Le Flore, OK
	40173	NA	Adult	97.00	76.50	1981	Shelby, TN
	40175	M	Adult	99.70	78.90	1984	Shelby, TN

*Ontogenetic stage inferred from epiphyseal fusion as described above.

**Fig 1:**
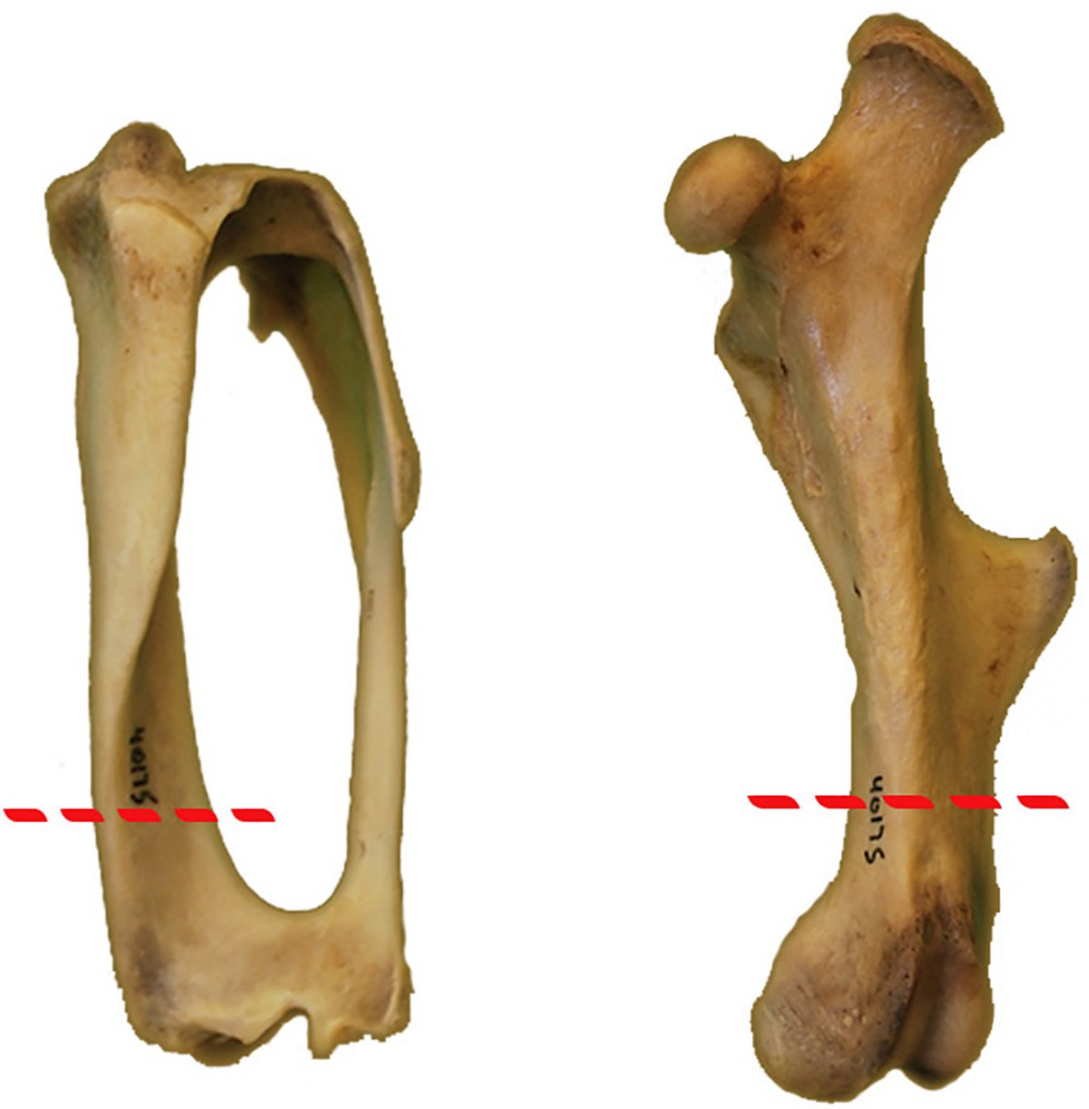
Location of sectioning in tibiae (left) and femora (right) of *Dasypus novemcinctus*.

Femora were cut transversely, proximal to the third trochanter (Fig 1) using a Dremel rotary tool equipped with a carbon steel brush tip. The distal ‘halves’ of femora and full tibiae/fibulae complexes were prepared using techniques outlined in Schweitzer *et al*. [59]. The third trochanter arises on the proximal shaft of the femur in armadillos and extends distally below the mid-diaphyseal region. Thus, two serial transverse sections of the embedded femora were removed distal to the third trochanter in an attempt to minimize interference from growth of the trochanter. Extension of bony processes such as trochanters and tubercles typically requires increased bone deposition in the region of those processes and are not representative of overall bone growth. Two transverse sections were removed from the diaphysis of the embedded tibiae distal to the apex of the tibial crest (Fig 1). Two thin sections of approximately 1.4mm thickness were removed from each element and mounted to plastic slides. Slides were ground and polished to a thickness between 70μm and 100μm using a series of carbide grit papers on a lapidary wheel. After polishing, one slide from each element was stained with toluidine blue following protocol by Osborne and Curtis [60], except in step 4 staining in 1% toluidine blue solution was performed for 5 minutes and in step 6 differentiation in 70% EtOH was completed in a range of 3–5 dips, depending on the rate of color change. Each stained thin section was then protected with a cover-slip to ensure the long-term preservation of the thin section. Toluidine blue was used to increase visibility and differentiation of lines of arrested growth (LAGs) and cement lines of secondary osteons. Toluidine blue staining was completed by the Montana Veterinary Diagnostic Laboratory in Bozeman, Montana, USA (UTCM 801, UTCM 802, and UTCM 1557) and the Woodward Ballard Paleohistology Lab at Oklahoma State University-Center for Health Sciences in Tulsa, OK, USA. Thin section analyses were performed at the Woodward Ballard Paleohistology Lab on the Oklahoma State University Center for Health Sciences campus (Tulsa, OK). Thin sections were examined using a Nikon Ni-U Eclipse polarized transmitted light microscope in linearly (plane) polarized, cross-polarized, and circularly polarized light, and photographs were taken using a Nikon DS-Ri-2 camera mounted to a Nikon Ni-U Eclipse polarizing microscope. Composite stitching of full slide scans was completed automatically by Nikon NIS Elements: Documentation. Figures at high resolution are available at morphobank.org (Project P2750).

All descriptions of bone microstructure are based on terminology in Francillon-Viellot *et al*. [61] and Enlow [40] and are defined here. We stress that the organization of mineralized collagen fibers is on a continuum. Therefore, intermediate states of organization exist between the defined terms below. For example, tissue organization can be more organized than woven tissue but less organized than parallel-fibered tissue. Woven bone tissue consists of unorganized collagen fibers and is regionally isotropic in cross polarized light, in both transverse and longitudinal sections. Osteocyte lacunae in woven bone often appear “plump” (i.e. rounded) or irregularly shaped without clear major axis orientation. Lamellar tissue is composed of highly organized collagen fibers appearing as a plywood pattern and is anisotropic with high birefringence in cross polarized light, when fibers are arranged parallel to the plane of section; when perpendicular to the plane of section, they appear isotropic. However, recent analysis of lamellar tissue found collagen fibers interweaving within arrays composing a single lamella, despite the plywood patterning seen at a more macroscopic level [62]. Osteocyte lacunae within lamellar tissue are often sparse and appear elongated or flattened within the meshwork of lamellar tissue fibers, parallel to the plane of section [61]. Parallel-fibered tissue is anisotropic in cross polarized light when collagen fibers are parallel to the plane of section and appear isotropic if oriented perpendicular to the plane of section. Parallel-fibered tissue consists of collagen fibers arranged in parallel, often with elongated osteocyte lacunae. The mineralized collagen fibers in parallel-fibered tissue are arranged more or less parallel to each other, and so this tissue is more organized than woven tissue but less organized than lamellar tissue. However, parallel-fibered tissue can vary in regards to the degree of collagen fiber organization. As noted by Francillon-Viellot *et al*. [61], ‘…there are intermediate categories between [parallel-fibered bone] and both woven bone and lamellar bone, as expressed in the level of the fibrillar arrangement’. We use the term “loosely arranged parallel-fibered” to describe tissue that appears intermediate to parallel-fibered tissue and woven tissue. Tissue organization can be used to determine relative growth rates, and daily apposition rates have been observed for lamellar (≤ 1μm/day), parallel-fibered (1 μm/day), and woven tissue (5–171 μm/day) [22, 28, 63–67].

Compacted coarse cancellous bone (CCCB) is a term applied to bone tissue formed when new bone fills in the spaces between existing trabeculae, subsequently compacting the cancellous layer of bone [40]. CCCB is often formed of lamellar tissue deposited along trabecular surfaces, resulting in regions of both high and low birefringence in cross polarized light. Resulting features of the formation of CCCB can be irregularly shaped osteon-like formations, multitudes of tide lines throughout the tissue delineating un-resorbed trabeculae from more recently deposited lamellar tissue, and islands of calcified cartilage if the CCCB is near trabeculae formed in the metaphyseal region at an earlier ontogenetic stage, which has since migrated into the diaphysis through the process of diaphyseal elongation [40]. Islands of calcified cartilage were identified through toluidine staining and morphological consistencies with previous descriptions (see Fig 8.21 in [68]). Descriptions of tissue types follow these definitions unless noted otherwise.

Additional features include resorption lines, external fundamental system (EFS), and “metaplastic tissue”. Resorption lines result from deposition of tissue on a previously eroded surface. The resorption line often has a wavy appearance outlining previously existing Howship’s lacunae formed from osteoclast activity. An external fundamental system (EFS) is an outer cortical layer of well-organized tissue representing a slowing and eventual cessation of cortical growth and, thus, skeletal maturity [69]. An EFS is typically composed of avascular lamellar or parallel-fibered tissue with closely spaced growth marks, but can also be a thick annulus at the periosteal surface. ‘Metaplastic tissue’ described herein refers to tissue that is morphologically consistent with metaplastic hard tissues defined and described in Haines and Mohuiddin [70] (termed ‘metaplastic bone’) and Horner *et al*. [71]. Metaplastic hard tissues described below are likely the result of mineralizing tendinous fibers at a muscular attachment site (enthesis), termed intratendinous metaplastic tissue [72]. However, due to our limited sample size and singular sampling location we cannot eliminate a cartilaginous origin for the metaplastic tissue. We attempt to note the presence of metaplastic tissue throughout our sample, but the extent and full investigation of this mineralized tissue is the subject of a future study. Likewise, periosteal attachment fibers (e.g. Sharpey’s fibers), and associated fibrocyte lacunae, are found throughout our sample but will not be noted further and is beyond the scope of this paper.

## Results

### UTCM 802

**Fig 2:**
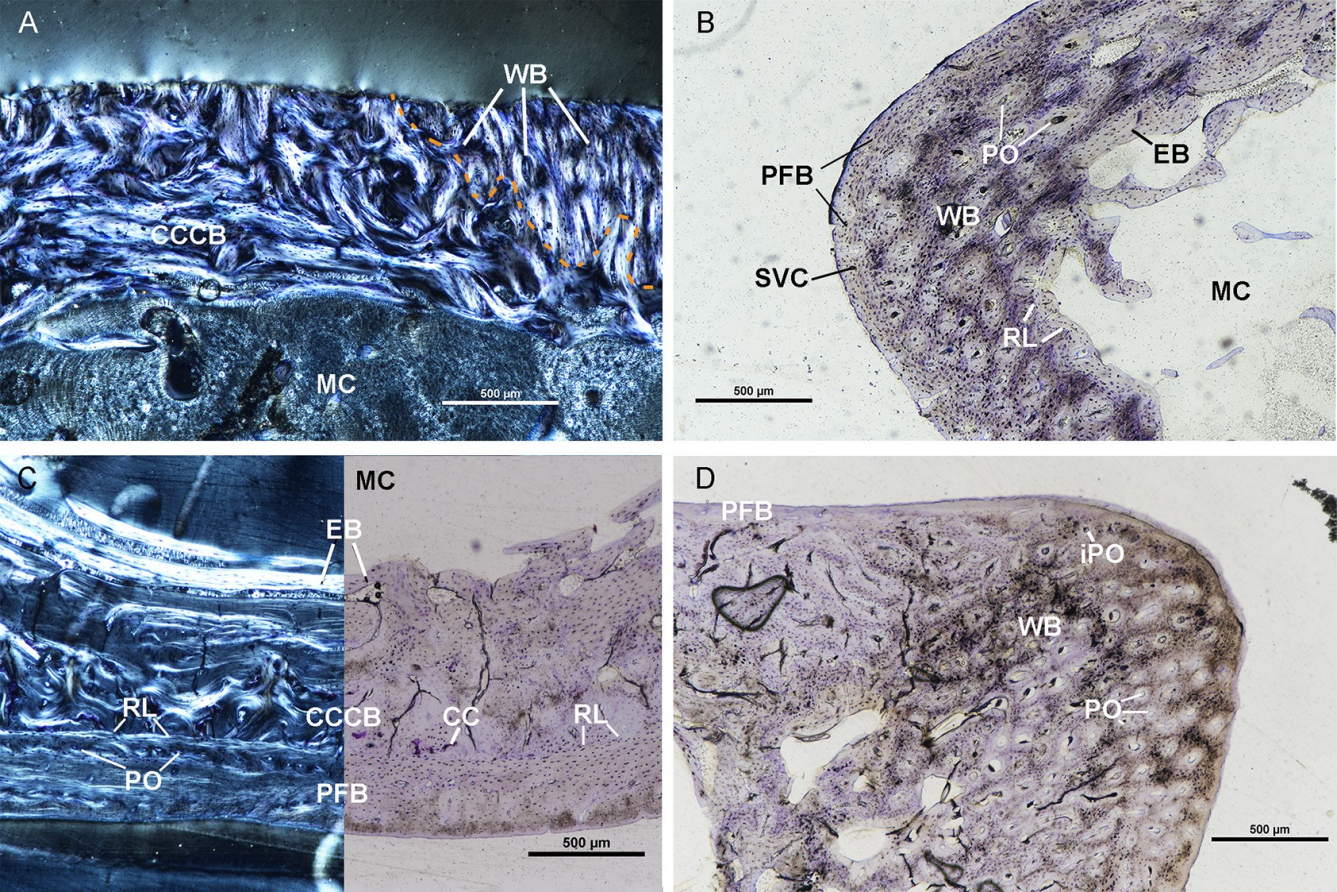
Osteohistological features of UTCM 802 femur and tibia. (A) Anterolateral side of femur in circularly polarized light. Cortex of the femur is CCCB medially (left of image) and transitions to woven bone laterally (right of image). Orange dotted line indicates resorption line separating the two tissue types. (B) Lateral corner of the femur of UTCM 802 in linearly polarized light is composed of three layers: endosteal bone lining parts of the medullary cavity, primary woven bone, and an outer cortex of loosely organized parallel-fibered bone. Primary osteons are present within the woven bone cortex and simple vascular canals within the outer cortex of parallel-fibered bone. (C) Posterior cortex of the tibia of UTCM 802 under circularly polarized light (left) and linearly polarized light (right). The endosteal bone is composed of lamellar tissue with radially oriented vascular canals. The mid-cortex on the posterior side is CCCB and regions of calcified cartilage (stained violet/purple). The outer cortex is parallel-fibered bone with simple vascular canals and primary osteons and is separated from the mid-cortex by a resorption line. (D) Antero-medial corner of the tibia of UTCM 802 under linearly polarized light. The cortex consists of woven bone with primary osteons and simple longitudinal vascular canals. An outer cortex of parallel-fibered bone along the anterior border is not complete around the medial corner and is separated from the mid-cortex by a resorption line. All sections stained with toluidine blue. Calcified cartilage–CC, Compacted coarse cancellous bone–CCCB, Endosteal bone–EB, Incipient primary osteon–iPO, Medullary cavity–MC, Parallel-fibered bone–PFB, Primary osteon–PO, Resorption line–RL, Simple vascular canal–SVC, Woven bone–WB.

The femur of UTCM 802 has an endosteal layer of lamellar tissue of varying thickness. This layer is thickest along the medial and posterior sides. Regional endosteal resorption of the endosteal layer was occurring at the time of death on the lateral side as revealed by the intermittent presence of scalloped pitting along the endosteal surface. Woven tissue with dense osteocyte lacunae comprises the mid-cortex of the lateral side of the femur with primary osteons and vascular canals oriented longitudinally and obliquely (Fig 2B). Few irregular secondary osteons are present in the woven tissue on the postero-lateral side. The mid-cortical woven tissue is separated from the endosteal layer by a resorption line indicating endosteal resorption of the woven tissue occurred prior to deposition of endosteal bone. Medially the woven tissue changes to CCCB as the major component of the cortex (Fig 2A). The CCCB typically extends to the periosteal surface on the medial, posterior, and anterior sides. The outermost cortex consists of loosely arranged parallel-fibered tissue on the lateral corner. In cross polarized light, this outer cortex has high birefringence postero-laterally, but also numerous plump osteocyte lacunae. The outer cortex lacks birefringence on the antero-lateral portion of the lateral corner, possibly due to a shift in fiber organization to woven tissue or a change in orientation of parallel-fibered tissue. Vascular canals are simple or surrounded by primary osteons and are oriented longitudinally, occurring within the outer cortex and are more numerous in the isotropic region. Periosteal resorption is indicated by intermittent scalloped pitting on the periosteal surface along the posterior, antero-medial, and antero-lateral sides of the femur.

The tibia of UTCM 802 has an endosteal layer of lamellar tissue that is continuous with few circumferentially oriented trabeculae (Fig 2C). In general, the mid-cortex of the tibia is composed of woven tissue and CCCB. Within the mid-cortex of woven tissue are resorption cavities, frequent simple vascular canals, incipient primary osteons, and primary osteons surrounding longitudinal vascular canals (Fig 2D). This mid-cortical woven bone extends to the periosteal surface on the medial side. The mid-cortex of the anterior, lateral, and posterior sides of the tibia is CCCB with pockets of calcified cartilage concentrated posteriorly and laterally (Fig 2C). The CCCB extends to the periosteal surface on portions of the lateral and anterior sides. The mid-cortex and endosteal layer of lamellar tissue are separated by a resorption line indicating endosteal resorption of the mid-cortex occurred prior to deposition of the inner lamellar tissue. An outer cortex of woven tissue with longitudinal vascular canals surrounded by primary osteons, as well as simple vascular canals, is visible along a posterior portion of the lateral side of the tibia. Along the posterior side, the outer cortex transitions into loosely organized parallel-fibered tissue with longitudinal vascular canals surrounded by primary osteons (Fig 2D). Anteriorly, the outer cortex is parallel-fibered tissue with high birefringence but mostly plump osteocyte lacunae, but the parallel-fibered tissue does not continue along the entire anterior side due to the aforementioned CCCB extending to the periosteal surface anteriorly. Along with the outer cortex of woven tissue, the parallel-fibered tissue is separated from the mid-cortex by a resorption line; thus, periosteal erosion of the mid-cortex occurred prior to deposition of the outer cortex.

### UTCM 801

**Fig 3:**
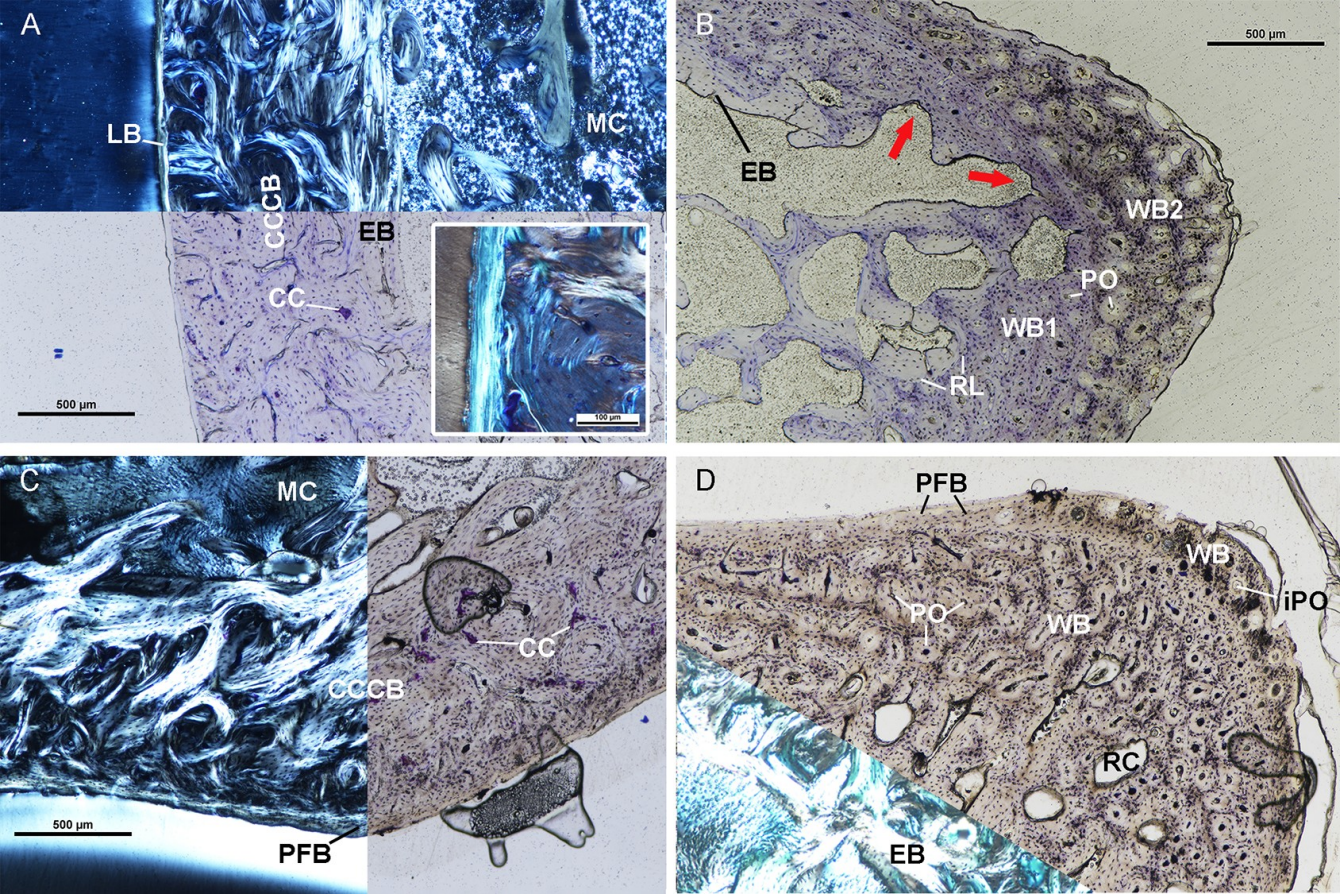
Osteohistological features of UTCM 801 femur and tibia. (A) Posterior cortex of the femur of UTCM 801 under circularly polarized light (top) and linearly polarized light (bottom). The innermost cortex is endosteal bone composed of lamellar tissue. The mid-cortex of the posterior side is composed of CCCB with regions of calcified cartilage. The outer cortex is avascular lamellar bone. Inset is outer cortex of lamellar tissue under circularly polarized light at higher magnification. (B) Lateral corner of the femur of UTCM 801 under linearly polarized light. The cortex is composed of three regions: endosteal bone along the medullary cavity, an older primary woven bone (WB1), and an outer cortex of fibrous primary woven bone (WB2). The endosteal bone and older region of woven bone were being resorbed (red arrows) at the time of death. Both regions of woven bone have primary osteons. WB1 is separated from the endosteal bone by a resorption line. (C) Posterior cortex of the tibia of UTCM 801under circularly polarized light (left) and linearly polarized light (right). The mid-cortex is composed of compacted coarse cancellous bone with regions of calcified cartilage and an outer cortex of parallel-fibered bone. (D) Antero-medial corner of the tibia of UTCM 801 in linearly polarized light (top right) and circularly polarized light (bottom left). The innermost cortex is composed of endosteal bone with numerous resorption cavities. The mid-cortex is woven bone with primary osteons surrounding longitudinally oriented vascular canals. Resorption cavities are present in the endosteal bone and mid-cortex. The outer cortex is woven bone with simple vascular canals at the antero-medial corner. Woven bone transitions to parallel-fibered bone anteriorly and medially. All sections stained with toluidine blue. Calcified cartilage–CC, Compacted coarse cancellous bone–CCCB, Endosteal bone–EB, Incipient primary osteon–iPO, Lamellar bone–LB, Medullary cavity–MC, Parallel-fibered bone–PFB, Primary osteon–PO, Resorption cavity–RC, Resorption line—RL, Woven bone–WB.

The femur of UTCM 801 has an incomplete endosteal layer of lamellar tissue (Fig 3A and 3B). At the lateral corner the lamellar endosteal layer and innermost cortex is resorbed due to medullary drift (Fig 3B). The lamellar tissue is birefringent in cross polarized light on the medial side. Elsewhere, the endosteal layer contains primarily plump osteocyte lacunae and lacks birefringence, possibly due to a longitudinal orientation of collagen fibers. The endosteal layer has few simple vascular canals oriented longitudinally and radially. Overall the lamellar endosteal layer in UTCM 801 is not as extensive in thickness as in the femur of UTCM 802. The cortex of the femur is primarily CCCB and woven tissue. Similar to UTCM 802, woven tissue with numerous primary osteons and incipient primary osteons makes up the mid-cortex and outer cortex of the lateral corner and transitions to CCCB on the antero-medial and postero-medial sides. The woven tissue increases vascularity and lacunar density towards the periosteal surface. Primary osteons are present in the woven tissue and surround longitudinal vascular canals (Fig 3B). The woven tissue has resorption cavities and scalloping along the endosteal surface and is adjacent to the medullary cavity at the lateral corner (Fig 3B). Elsewhere a resorption line separates the woven tissue from the endosteal lamellar tissue similar to UTCM 802. The mid-cortex of the medial side of the femur is CCCB (Fig 3A). The CCCB continues around the anterior and posterior sides where the mid-cortex transitions to woven tissue laterally. Medially, the CCCB is separated from the endosteal layer by a resorption line indicating endosteal resorption of CCCB occurred prior to deposition of endosteal bone. The CCCB extends out to the periosteal surface along a stretch of the posterior side where woven tissue is not present. Calcified cartilage is found throughout the region of CCCB and identified via toluidine blue staining (Fig 3A). Unlike UTCM 802, an outer cortex composed of varying tissue organizations is present on the medial side and regions of the anterior and posterior sides of the femur, but absent from the apex of the lateral corner where the aforementioned woven tissue is present. The outer cortex is parallel-fibered postero-laterally with both plump and elongated osteocyte lacunae. The outer cortex is lamellar tissue on the postero-medial and medial side of the femur with very few simple longitudinal vascular canals (Fig 3A). Osteocyte lacunae are scarce in the outer lamellar tissue on the postero-medial and medial sides. The outer cortex lacks birefringence in cross polarized light postero-medially but is birefringent along most of the medial side. The outer cortex is disorganized woven tissue on the anterior side. The shift in organization is accompanied by an increase in longitudinal vascular canals and primary osteons. The outer cortex is parallel-fibered tissue antero-laterally, is only somewhat birefringent, and contains primarily plump osteocyte lacunae.

The endosteal surface of the tibia of UTCM 801 is composed of cancellous tissue in the form of a dense network of trabeculae. Resorption of the trabeculae and endosteal bone on the medial side is evident by scalloping along the surfaces of the trabeculae and resorption cavities within the endosteal bone layer. The mid-cortex is composed of woven tissue and CCCB. Similar to UTCM 802, the mid-cortex is CCCB along the anterior, posterior, and lateral sides (Fig 3C). The CCCB extends to the periosteal surface antero-laterally. Regions of calcified cartilage within the CCCB are concentrated laterally and posteriorly (Fig 3C). The mid-cortex of the medial side is also similar to UTCM 802 in that it is composed of woven tissue. Large resorption cavities are present within the woven tissue as are longitudinally oriented vascular canals incased by primary osteons (Fig 3D). There is an outer cortex of avascular parallel-fibered tissue with plump osteocyte lacunae along the posterior length of the lateral side (Fig 3C). The outer cortex becomes woven at the postero-lateral corner with longitudinally oriented vascular canals surrounded by primary osteons, and simple vascular canals. The outer cortex transitions to birefringent parallel-fibered tissue with plump osteocyte lacunae on the posterior side. Medially the outer cortex is woven with longitudinally oriented primary osteons and simple vascular canals at the medial corner (Fig 3D).

### OMNH 39188

**Fig 4:**
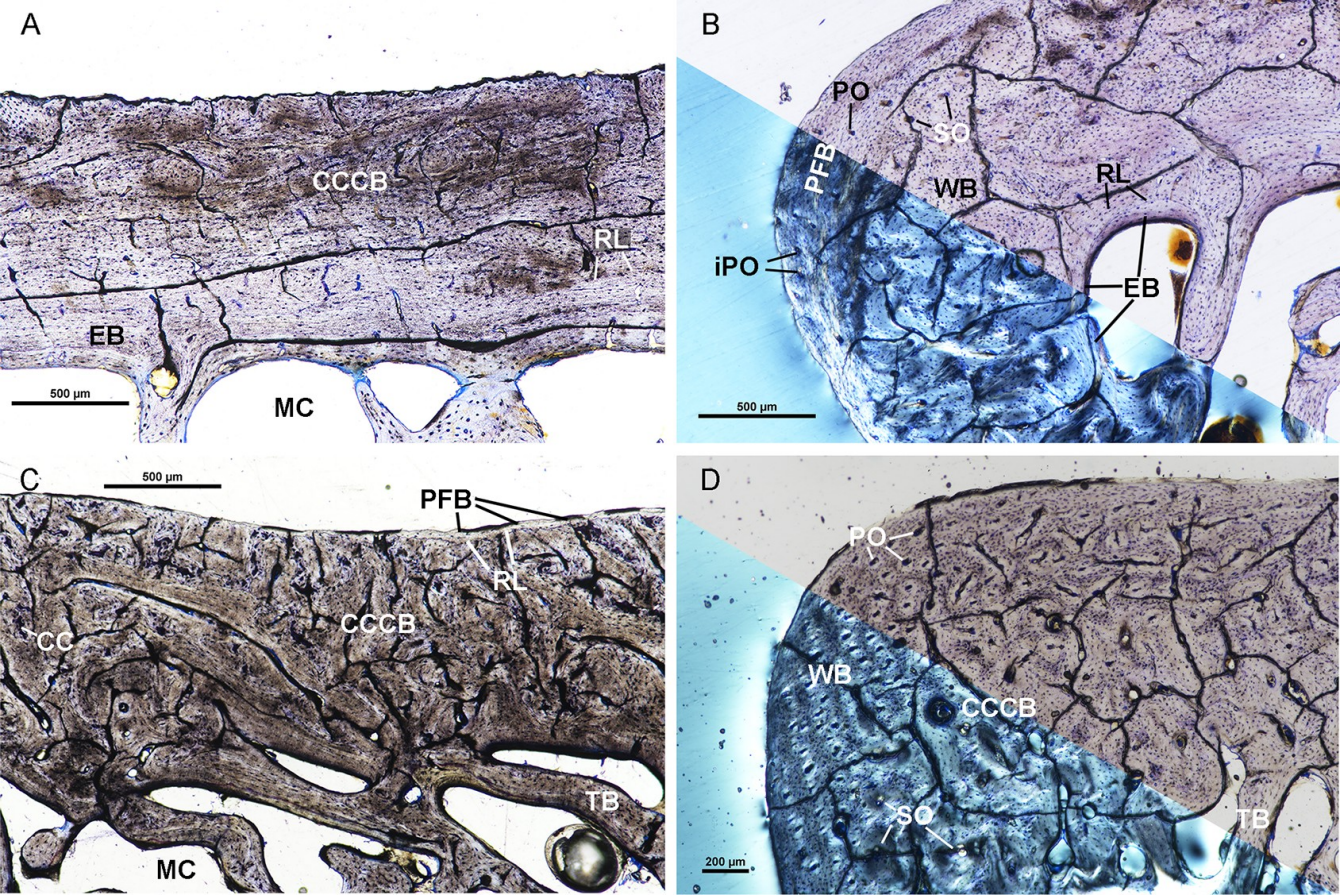
Osteohistological features of OMNH 39188. (A) Posterior cortex of the femur of OMNH 39188, under linearly polarized light, is composed of endosteal bone and CCCB. The endosteal bone has few radial vascular canals. The outer cortex of CCCB is separated from the inner endosteal bone by a resorption line. (B) Lateral corner of the femur of OMNH 39188 under circularly polarized light (bottom) and linearly polarized light (top). The lateral corner is composed of an inner cortex of endosteal bone, woven bone in the mid-cortex, and an outer cortex of parallel-fibered bone. The mid-cortex has few secondary osteons and is separated from the endosteal bone by a resorption line. The outer cortex of parallel-fibered bone has simple longitudinal vascular canals and primary osteons surrounding longitudinally oriented vascular canals. (C) Posterior side of the tibia of OMNH 39188 under linearly polarized light. The innermost cortex is composed of cancellous trabecular bone. CCCB comprises the mid-cortex with regions of calcified cartilage. A thin outer cortex of parallel-fibered bone is separated from the mid-cortex by a resorption line. (D) Antero-medial corner of the tibia of OMNH 39188 under circularly polarized light (bottom) and linearly polarized light (top). The innermost cortex is cancellous trabecular bone. The mid-cortex is composed of CCCB with few secondary osteons indicating remodeling of the CCCB has occurred. The outer cortex is woven bone with primary osteons surrounding longitudinally oriented vascular canals. All sections stained with toluidine blue. Calcified cartilage–CC, Compacted coarse cancellous bone–CCCB, Endosteal bone–EB, Incipient primary osteon–iPO, Medullary cavity–MC, Parallel-fibered bone–PFB, Primary osteon–PO, Resorption line–RL, Secondary osteon–SO, Trabecular bone–TB, Woven bone–WB.

The femur of OMNH 39188 has an endosteal layer of lamellar tissue of varying thickness with vascular canals oriented longitudinally and radially (Fig 4A). The endosteal layer is birefringent under cross polarized light except for a region of the medial side. The endosteal layer composes most of the cortex on the posterior side and is relatively thicker than the endosteal layers of UTCM 801 and UTCM 802. The mid-cortex of the femur is primarily CCCB. However, the mid-cortex of the lateral corner is heavily remodeled woven tissue containing numerous secondary osteons (Fig 4B). The heavily remodeled woven tissue transitions to CCCB anteriorly and posteriorly without a resorption line delineating the two tissue types. Therefore, the heavily remodeled woven tissue at the lateral corner could be a continuation of mid-cortical CCCB. The CCCB extends to the periosteal surface on the posterior side similar to UTCM 801 (Fig 4A). CCCB is separated from the endosteal layer by a resorption line. The outer cortex on the medial, anterior, and lateral sides of the femur is composed of varying tissue organizations and is generally thicker than the outer cortex of various organizations present in UTCM 801. Laterally, the outer cortex is dis-organized parallel-fibered tissue with longitudinally oriented incipient primary osteons, and primary osteons (Fig 4B). The outer cortex is well organized parallel-fibered tissue along the anterior and medial sides with regions of disorganized fibers and primary osteons. The outer cortex on the anterior side is highly birefringent in cross polarized light, whereas the outer cortex on the medial side is isotropic. The outer cortex at the postero-medial corner is avascular lamellar tissue. The outer cortex is separated from the mid-cortex by a resorption line, indicating periosteal resorption of the mid-cortical tissues occurred prior to deposition of the outer cortex.

The tibia of OMNH 39188 has an inner cortex composed of cancellous tissue as in the tibia of UTCM 801 (Fig 4C). The mid-cortex is CCCB with regions of calcified cartilage concentrated on the anterior and medial sides of the tibia. The CCCB extends out to the periosteal surface on the medial side. Elsewhere, the outer cortex is composed of woven and parallel-fibered tissue. The outer cortex is parallel-fibered tissue along most of the anterior and posterior sides (Fig 4C). The outer cortex transitions to woven tissue with longitudinally oriented vascular canals surrounded by primary osteons at the postero-lateral corner. The outer cortex has woven tissue with longitudinally oriented vascular canals surrounded by primary osteons, as well as secondary osteons, at the medial corner (Fig 4D). Secondary osteons are concentrated in the inner portion of the outer cortex at the medial corner.

### UTCM 1557

**Fig 5:**
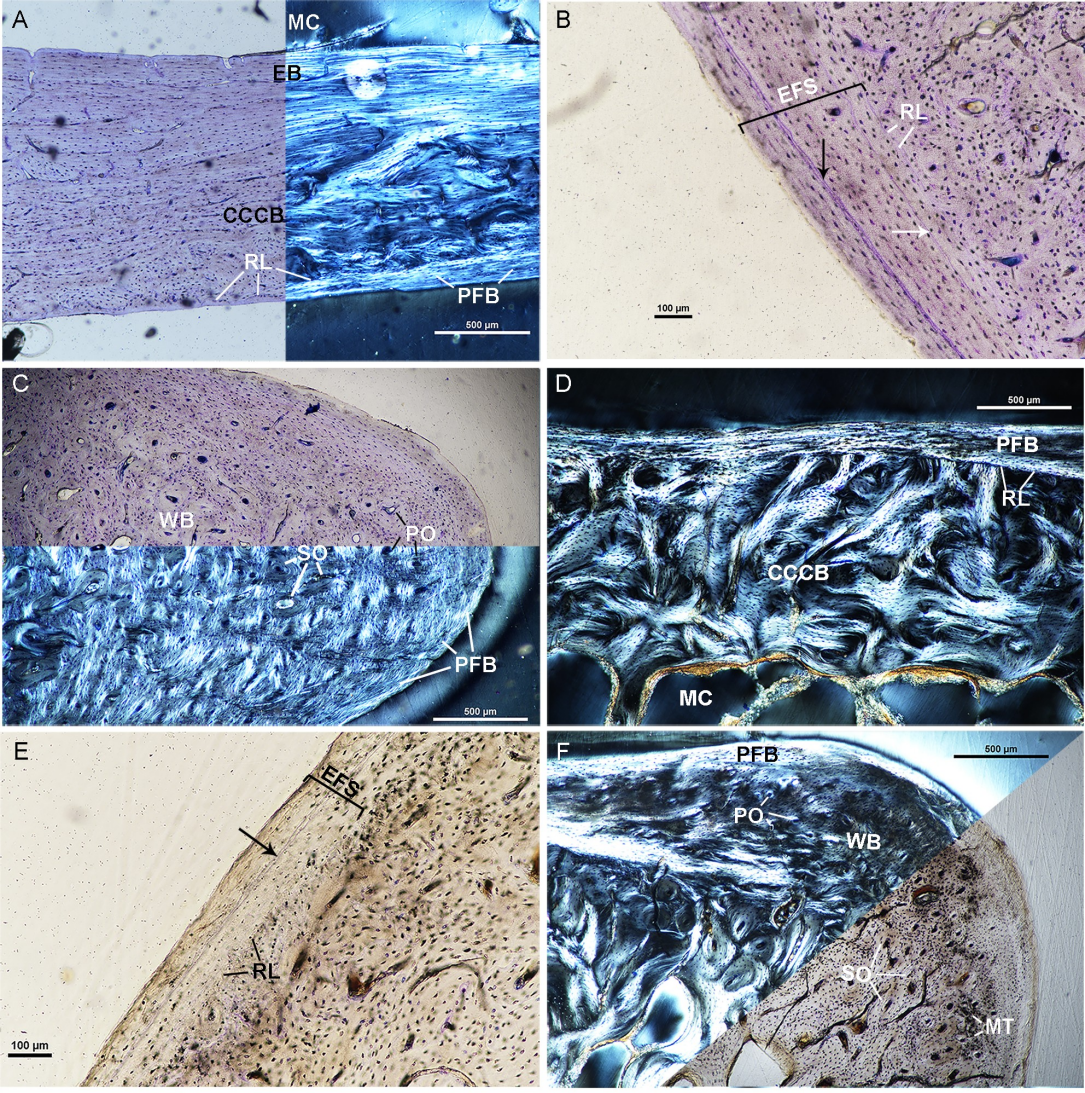
Osteohistological features of UTCM 1557 femur and tibia. (A) Posterior cortex of the femur of UTCM 1557 under linearly polarized light (left) and circularly polarized light (right). The cortex is composed of an inner cortex of endosteal bone, mid-cortex of CCCB, and outer cortex of avascular parallel-fibered bone. The outer cortex is separated from the mid-cortex by a resorption line. (B) Outer cortex of the antero-lateral side of the femur of UTCM 1557 under linearly polarized light. The mid-cortex is woven bone with secondary osteons and primary osteons. The outer cortex is avascular lamellar bone with a double LAG (black arrow), representing an external fundamental system. The outer cortex is separated from the mid-cortex by a resorption line. A faint line, possibly an additional LAG, is present (white arrow), though this line is not continuous beyond the region imaged. (C) Lateral corner of the femur of UTCM 1557 under linearly polarized light (top) and circularly polarized light (bottom). The mid-cortex is composed of remodeled woven tissue with primary and secondary osteons. The outer cortex at the lateral corner is avascular parallel-fibered tissue. (D) Anterior side of the tibia of UTCM 1557 under circularly polarized light. The mid-cortex on the anterior side is CCCB. The outer cortex is parallel-fibered bone of varying thickness. The outer cortex is separated from the mid-cortex by a resorption line. (E) The outer cortex of the lateral side of the tibia under linearly polarized light. Here, the outer cortex is highly organized parallel-fibered bone with a double LAG present (arrow). The outer cortex is separated from the mid-cortex by a resorption line. (F) Antero-medial corner of the tibia of UTCM 1557 under circularly polarized light (top) and linearly polarized light (bottom). The mid-cortex is composed of woven bone with primary and secondary osteons. Regions of metaplastic tissue are present posteriorly at the corner. The outer cortex is highly organized parallel-fibered bone. All sections stained with toluidine blue. Compacted coarse cancellous bone–CCCB, Endosteal bone–EB, External fundamental system–EFS, Lamellar bone–LB, Growth marks–black arrows, Medullary cavity–MC, Metaplastic tissue–MT, Parallel-fibered bone–PFB, Primary osteon–PO, Resorption line—RL, Secondary osteon–SO, Woven bone–WB.

The femur of UTCM 1557 has an endosteal layer of lamellar tissue of varying thickness. Few radial vascular canals are present in the endosteal layer (Fig 5A). The lamellar endosteal layer is thinnest at the postero-medial and lateral corners. The woven mid-cortex has numerous primary and secondary osteons. Few resorption cavities are present and were in the process of centripetal infilling to form secondary osteons (Fig 5C). The woven tissue transitions to CCCB on the antero-lateral and postero-lateral sides. The CCCB contains a few regions of calcified cartilage. Secondary osteons are present at the antero-medial corner and remodeled regions of the CCCB. Similar to previous specimens, the CCCB is separated from the inner endosteal layer by a resorption line. The outer cortex of UTCM 1557 is primarily avascular lamellar tissue that we interpret as an EFS (Fig 5B). The outer cortex is birefringent in cross polarized light except for the anterior side and postero-medial corner. The outer cortex becomes less organized, transitioning to a more vascular parallel-fibered tissue laterally (Fig 5C). Longitudinally oriented simple vascular canals and vascular canals within primary osteons are present in the outer cortex on the antero-lateral and postero-lateral sides. The outer cortex has two hyper-mineralized lines made evident by toluidine blue staining at the anterior, antero-lateral, and postero-lateral sides (Fig 5B). These two lines are likely a double-LAG and represent a single cessation of growth event [73–74]. The outer cortex is separated from the mid-cortical CCCB by a resorption line (Fig 5B).

The innermost tissue of the tibia of UTCM 1557 is composed primarily of cancellous bone similar to previously described tibiae above. The mid-cortex of UTCM 1557 is CCCB and woven tissue with a few secondary osteons concentrated on the medial side (Fig 5F). Regions of calcified cartilage in the CCCB are primarily located on the posterior side of the tibia, though several small areas are found medially and anteriorly. The tibia has an outer cortex of parallel-fibered tissue and woven tissue, being a thin region of parallel-fibered tissue around a majority of the lateral and anterior sides, but thicker on the posterior and medial sides (Fig 5D). The outer cortex of the posterior side is alternating regions of woven and parallel-fibered tissue. Longitudinally oriented vascular canals within primary osteons and simple vascular canals are found within the woven tissue components of the outer cortex. The outer cortex transitions to parallel-fibered tissue medially until transitioning to a thin region of avascular parallel-fibered tissue at the medial corner (Fig 5F). Two growth marks, potentially a double LAG, are present in the outer cortex near the medial corner (Fig 5E). These growth marks do not continue around the entire circumference of the tibia. Osteocyte lacunae in the outer cortex at the medial corner are plump and the tissue is isotropic, likely due to collagen fibers running in parallel longitudinally. The outer cortex is separated from the CCCB by a resorption line (Fig 5E).

### OMNH 40173

**Fig 6:**
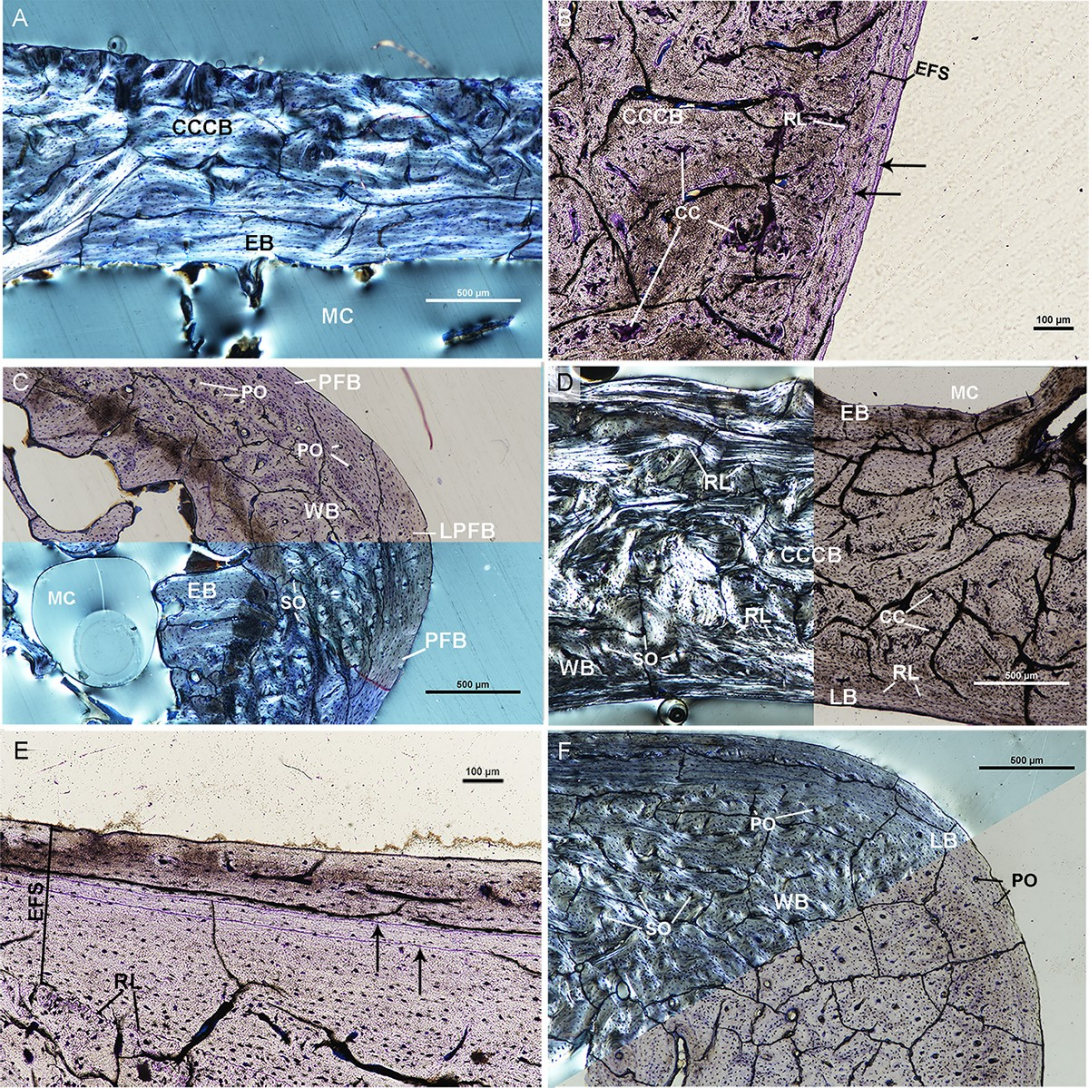
Osteohistological features of OMNH 40173. (A) Anterior side of the femur of OMNH 40173 under circularly polarized light. The cortex on the anterior side is composed of an inner cortex of endosteal bone and outer cortex of CCCB. (B) The postero-lateral side of the femur of OMNH 40173 under linearly polarized light. The mid-cortex is CCCB with regions of calcified cartilage. The outer cortex is interpreted as an incipient EFS as the EFS does not continue around the entire circumference of the femur. Two LAGs (arrows) are present within the EFS. (C) Lateral corner of the femur of OMNH 40173 under linearly polarized light (top) and circularly polarized light (bottom). The lateral corner has an inner cortex of endosteal bone. The mid-cortex is woven bone with primary and secondary osteons. The outer cortex is parallel-fibered tissue. The parallel-fibered tissue at the apex of the lateral corner is oriented longitudinally in relation to the bone shaft. The outer cortex has longitudinally oriented simple vascular canals. (D) The posterior cortex of the tibia of OMNH 40173 under circularly polarized light (left) and linearly polarized light (right). The posterior side has an inner cortex of endosteal lamellar bone. The mid-cortex is CCCB with regions of calcified cartilage and woven bone. The mid-cortical CCCB is separated from both the inner cortex and mid-cortical woven bone by resorption lines. The woven bone mid-cortex has secondary osteons and is separated from the outer cortex of avascular lamellar bone by a resorption line. (E) The anterior side of the tibia of OMNH 40173 under linearly polarized light. The outer cortex of lamellar bone is separated from the mid-cortex by a resorption line. The outer cortex has two LAGs (arrows) and is potentially an EFS. Note–there is a circumferential fracture within the outer cortex near the outer portion of the double-LAG. (F) The antero-medial corner of the tibia of OMNH 40173 under circularly polarized light (top) and linearly polarized light (bottom). The mid-cortex is woven bone at the corner with secondary osteons and primary osteons. The outer cortex is lamellar bone with few simple vascular canals. All sections stained with toluidine blue. Calcified cartilage–CC, Compacted coarse cancellous bone–CCCB, Endosteal bone–EB, External fundamental system–EFS, Lamellar bone–LB, Lines of arrested growth–black arrows, Medullary cavity–MC, Parallel-fibered bone–PFB, Primary osteon–PO, Resorption line–RL, Secondary osteon–SO, Woven bone–WB.

The femur of OMNH 40173 has an incomplete endosteal layer of avascular lamellar tissue (Fig 6A). Resorption of the endosteal layer is intermittent on the lateral, anterior, and medial sides. Consistent with previous descriptions above, the mid-cortex of the femur is CCCB and woven tissue (Fig 6A–6C). The woven tissue is present on the lateral side and continues medially along the anterior and posterior sides until it transitions into CCCB. The woven tissue has longitudinal vascular canals within primary osteons, and secondary osteons (Fig 6C). Resorption lines on the endosteal surface of the woven tissue indicates endosteal resorption of the woven tissue prior to deposition of the endosteal tissue. The outer cortex of the femur is composed of CCCB anteriorly and highly organized parallel-fibered tissue. The outer cortical parallel-fibered tissue is birefringent antero-laterally and postero-laterally but lacks birefringence at the apex of the lateral corner. We identify this is due to a shift in fiber direction, where the parallel-fibered tissue is oriented perpendicular to the plane of section at the apex and parallel to the plane of section elsewhere. (Fig 6A–6C). The parallel-fibered tissue component is likely the beginning of an EFS that is incomplete around the circumference of the bone. Vascularity in the outer cortex of parallel-fibered tissue consists of scarce primary osteons and longitudinally oriented simple vascular canals. There is an increase in vascularity in the outer cortex of the lateral corner. As in UTCM 1557, two hyper-mineralized lines are present in regions of the outer cortex. Unlike UTCM 1557, these are interpreted as two distinct LAGs due to spacing between the two hyper-mineralized lines (Fig 6B).

The trabecular network in the tibia of OMNH 40173 is less developed within the inner cortex than in the previous tibiae. The tibia has an endosteal layer of lamellar tissue and a mid-cortex of CCCB and woven tissue (Fig 6D). CCCB composes the entire mid-cortex of the anterior side. The CCCB extends out to the periosteal surface on the anterior portion of the lateral side. CCCB then composes the internal portion of the mid-cortex on the posterior side. Regions of calcified cartilage within the CCCB are primarily found at the postero-lateral corner. Secondary osteons are also found within the CCCB. The CCCB is separated from the inner lamellar tissue by a resorption line. A layer of woven tissue with interstitial parallel-fibered tissue was deposited along the previously eroded periosteal surface of the CCCB along the posterior side of the tibia (Fig 6D). The woven tissue component of the mid-cortex has numerous longitudinally oriented vascular canals within primary osteons, and secondary osteons. Secondary osteons are concentrated closer to the medullary cavity, similar to OMNH 39188. The outer cortex of the tibia on the anterior, medial, posterior sides, and the posterior portion of the lateral side is a combination of lamellar and parallel-fibered tissue. The outer cortex is parallel-fibered at the antero-lateral corner, medial corner, and postero-medial corner, and, similar to the femur, is interpreted as an incipient EFS with two LAGs (Fig 6E). Simple longitudinal vascular canals are scarce but present in the parallel-fibered regions of the outer cortex. Elsewhere the outer cortex is composed of highly organized lamellar tissue (Fig 6F). The outer cortex is separated from the mid-cortical CCCB by a resorption line.

### OMNH 40175

**Fig 7:**
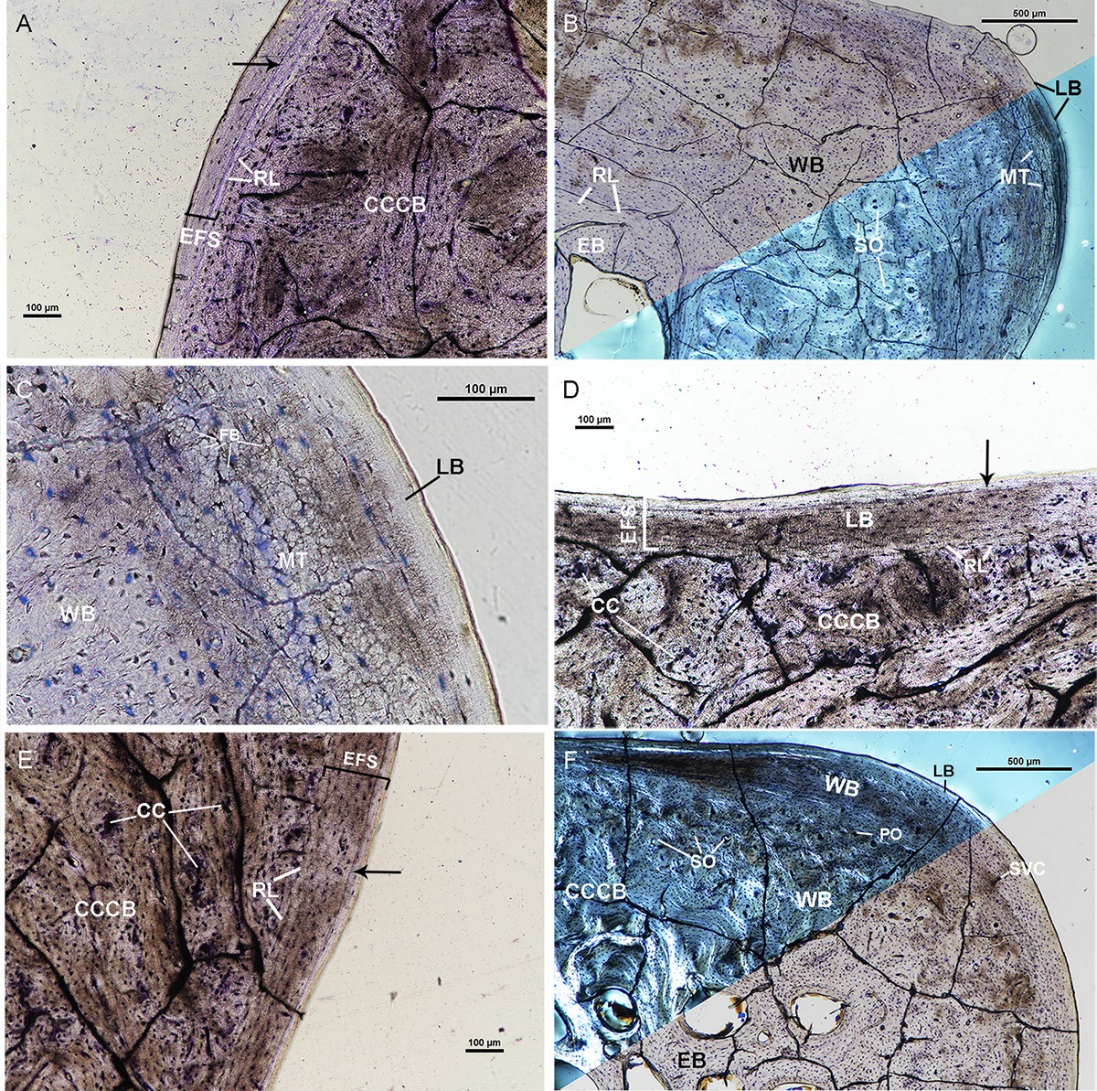
Osteohistological features of OMNH 40175 femur and tibia. (A)The antero-medial corner of the femur of OMNH 40175 under linearly polarized light. The mid-cortex is CCCB and the outer cortex is an external fundamental system composed of lamellar bone with a double LAG present (arrow). The outer cortex thins posteriorly and is separated from the mid-cortex by a resorption line. (B) Lateral corner of the femur of OMNH 40175 under linearly polarized light (top) and circularly polarized light (bottom). The mid-cortex of the lateral corner is woven bone with secondary osteons and regions of metaplastic tissue. The lateral corner has a thin outer cortex of avascular lamellar bone. (C) High magnification of the lateral corner of the femur under linearly polarized light to highlight metaplastic tissue. Metaplastic tissue is composed of fiber bundles oriented longitudinally often giving the appearance of osteocyte lacunae. (D) Posterior side of the tibia of OMNH 40173 under linearly polarized light. The mid-cortex is composed of CCCB with regions of calcified cartilage. The outer cortex on the posterior side is lamellar bone with one LAG present (arrow). The outer cortex is separated from the mid-cortex by a resorption line. (E) Medial side of the tibia showing the outer cortex of lamellar bone with one LAG (arrow). This outer cortex is interpreted as an EFS. (F) Antero-medial corner of the tibia under circularly polarized light (top) and linearly polarized light (bottom). The innermost cortex is endosteal lamellar bone. The mid-cortex is composed of CCCB and woven bone. CCCB composes the innermost portion of the mid-cortex and is separated from the endosteal bone by a resorption line. Woven bone comprises the outer portion of the mid-cortex and is partially remodeled with secondary osteons. The mid-cortical woven bone is less remodeled periosteally. The outer cortex is a thin region of lamellar bone at the antero-medial corner. All sections stained with toluidine blue. Calcified cartilage–CC, Compactedcoarse cancellous bone–CCCB, External fundamental system–EFS, Lamellar bone–LB, Line of arrested growth–black arrows, Metaplastic tissue–MT, Mineralized Fiber bundles–FB, Primary osteon–PO, Resorption line–RL, Secondary osteon–SO, Woven bone–WB.

The femur of OMNH 40175 has an endosteal layer of lamellar tissue. The mid-cortex is composed of remodeled woven tissue and CCCB, similar to OMNH 40173, UTCM 1557, and OMNH 39188 (Fig 7A and 7B). Woven tissue with interstitial metaplastic tissue and numerous secondary osteons composes the mid-cortex laterally (Fig 7B). The remodeled woven tissue transitions to CCCB medially along the anterior and posterior sides. As in other femora, a resorption line separates the mid-cortex and the endosteal bone indicating endosteal resorption of the mid-cortical tissues occurred prior to deposition of the endosteal layer. The outer cortex of lamellar and woven tissue is present around the circumference of the femur. The outer cortex of the lateral corner is composed of woven tissue with a large metaplastic tissue presence (Fig 7B and 7C). A few simple longitudinal vascular canals and secondary osteons are in the outer cortex of woven tissue. Elsewhere, the outer cortex is composed of avascular lamellar tissue and is an EFS (Fig 7A). Similar to UTCM 1557 and OMNH 40173, the outer cortex has two hyper-mineralized lines that are potentially the result of a single growth hiatus. A resorption line separates the outer cortex from the mid-cortex indicating periosteal resorption of the mid-cortex prior to the deposition of the outer cortex.

The tibia of OMNH 40175 has an extensive network of trabeculae similar to that found in OMNH 39188. The cancellous trabecular network comprises the inner cortex of the tibia. The mid-cortex of the tibia is composed of CCCB and woven tissue (Fig 7D–7F). CCCB is the innermost region of the mid-cortex and comprises the entire mid-cortex on the anterior, posterior, and lateral sides of the tibia (Fig 7D). Regions of calcified cartilage are found in the CCCB at the postero-lateral corner and secondary osteons are scarce within CCCB. A region of woven tissue is external to the CCCB at the medial side (Fig 7F). The woven tissue at the medial side is similar to previously described tibiae in that the woven tissue has longitudinally oriented vascular canals in primary osteons and secondary osteons concentrated internally closer to the medullary cavity. The outer cortex of the tibia is composed of lamellar tissue and woven tissue. The outer cortex is an EFS, with a single LAG, composed of avascular lamellar tissue on the lateral side and most of the anterior and posterior sides (Fig 7D and 7E). The outer cortex at the postero-medial region is comprised of woven tissue and lamellar tissue. The woven tissue in this region has longitudinally oriented vascular canals in primary osteons and simple vascular canals. The outer cortex at the medial side is weakly birefringent in cross-polarized light, has plump osteocyte lacunae, and longitudinally oriented vascular canals in primary osteons.

## Discussion

This study marks the first ontogenetic analysis, albeit a limited ontogenetic series, of bone microstructure changes in the long bones of the nine-banded armadillo, *D*. *novemcinctus*. Our smallest sampled femur, UTCM 802 (femoral length = 77.20 mm), has endosteal resorption lines within the cortex indicating moments of cortical drift. Endosteal resorption lines are likewise found throughout our entire sample of femora. Resorption cavities within the cortex are present in all individuals sampled, decreasing in frequency in larger individuals. Resorption cavities are more numerous throughout the cortex in the lateral corner of the femur, where resorption and deposition occur more rapidly due to the close proximity of the third trochanter. The mid-cortex of *D*. *novemcinctus* is composed mostly of CCCB with regions of primary woven tissue. Primary bone is resorbed both endosteally and periosteally throughout the femur, with the exception of the lateral corner, where only endosteal resorption occurred. All individuals have woven bone with longitudinal vascular canals in the lateral region and the number of secondary osteons in the lateral region increases through ontogeny. Vascularity in the lateral corner decreases in larger individuals and shifts to more longitudinally oriented canals within primary and secondary osteons. CCCB is formed from the infilling of cancellous bone, such as trabeculae, as the bone undergoes cortical drift. The extent of CCCB within the cortex increases throughout ontogeny in the femora of *D*. *novemcinctus*. An external fundamental system composed of poorly vascularized lamellar and parallel-fibered bone is deposited intermittently on the periosteally eroded surface of the femur in the larger individuals sampled. The EFS appears to be deposited first along the postero-medial and antero-medial corners. Deposition of the EFS then continues on the medial side and antero-medial side before being deposited lastly along the posterior and lateral sides. The EFS is continuous around the entire circumference of femora from UTCM 1557 (femur length = 95.59mm) and OMNH 40175 (femur length = 99.7mm), but not OMNH 40173 (femur length = 97mm). The incomplete nature of the EFS in OMNH 40173 indicates appositional growth was still occurring at the time of death. Lack of an EFS in OMNH 40173, despite a femoral length comparable to UTCM 1557 and OMNH 40175, could be due to individual variation or sexual dimorphism (see discussion below). The thickness of the cortex in the femur increases with size primarily as a result of thickening of the endosteal bone and deposition of the outer cortex of lamellar and parallel-fibered bone.

Bone microstructure in the tibia of *D*. *novemcinctus* is similar to that of the femur. Resorption lines within the cortex indicate endosteal and periosteal resorption events due to cortical drift. The smallest individual, UTCM 802, has a mid-cortex of primary woven tissue and CCCB. The general trend is the extent of primary woven bone decreases through ontogeny and CCCB increases (S1 Fig). The mid-cortex of larger individuals is composed of CCCB with regions of woven bone. Thickening of the cortex occurs in the tibia by formation of CCCB from the extensive network of trabeculae and deposition of lamellar and parallel-fibered bone along the periosteal surface. However, thickness of the CCCB and extensiveness of trabeculae are variable among individuals. The tibia of OMNH 40173 has fewer trabeculae than the other specimens and a well-defined endosteal layer of lamellar bone. An EFS of poorly vascularized lamellar and parallel-fibered bone appears first on the posterior side of the tibia in our limited ontogenetic series. The thickness of the EFS is highly variable between individuals and could be used as an indicator for relative age. The EFS then appears on the anterior side, followed by the lateral and then medial sides. Similar to the femora, an EFS is complete around the entire circumference of the tibiae from UTCM 1557 (tibia length = 78.23mm) and OMNH 40175 (tibia length = 78.9mm), but is incomplete in OMNH 40173 (tibia length = 76.5mm). Bone microstructural characteristics are summarized in Table 2.

**Table 2:** Osteohistological characteristics of sampled femora and tibiae.

	Specimen	Length (mm)	Epiphyseal Fusion	CCCB	No. of LAGs	EFS
**Femur**	UTCM 802	77.20	Incomplete	Yes	0	None
	UTCM 801	84.10	Incomplete	Yes	0	None
	OMNH 39188	91.50	Incomplete	Yes	0	None
	UTCM 1557	95.59	Nearly Fused	Yes	1	Complete
	OMNH 40173	97.00	Nearly Fused	Yes	2	Incomplete
	OMNH 40175	99.70	Complete	Yes	1	Complete
**Tibia**	UTCM 802	61.67	Incomplete	Yes	0	None
	UTCM 801	67.03	Incomplete	Yes	0	None
	OMNH 39188	74.00	Incomplete	Yes	0	None
	UTCM 1557	78.23	Nearly Fused	Yes	1	Complete
	OMNH 40173	76.50	Nearly Fused	Yes	2	Incomplete
	OMNH 40175	78.90	Complete	Yes	1	Complete

Tibiae and femora of *D*. *novemcinctus* undergo similar changes in growth according to our observations. Endosteal and periosteal erosion drives cortical drift with formation of CCCB. An outer cortex of lamellar and parallel-fibered bone is then deposited along eroded periosteal surfaces. However, thickness of the innermost cortex of endosteal bone is drastically different between the tibia and femur, with the femur typically having a much thicker endosteal layer of lamellar bone. This is likely due to the denser network of trabeculae found in the tibiae. An EFS is found in both tibiae and femora, but there appears to be intraspecific variation in regards to the extent and thickness of the EFS in both elements. For example, both the tibia and femur of OMNH 40173 have an incomplete outer cortical layer of well-organized tissue that we interpret as an incomplete, or incipient, EFS. The limited ontogenetic series suggests initial EFS deposition is discontinuous and regionally specific. Parfitt [58] suggested that fusion of the epiphyses is the result of growth cessation, thus, incomplete fusion would indicate skeletal maturity. Here, we found UTCM 1557 had nearly fused epiphyses but a complete EFS in both elements while OMNH 40173 had incomplete fusion and an incomplete EFS. EFS deposition seems to have occurred prior to epiphyseal fusion in UTCM 1557, but EFS deposition and epiphyseal fusion occurred concurrently in OMNH 40173. This suggests that further studies are needed to resolve the timing of epiphyseal fusion and diaphyseal osteohistological indicators of skeletal maturity.

The microstructural patterns of the nine-banded armadillo closely resemble those of the recently described *Orycteropus afer*, the aardvark [75]. Legendre and Botha-Brink [75] described the microstructure of various limb elements, including femora and tibiae, and found CCCB composing most of the cortex and a thin outer cortex of parallel-fibered and woven bone with few growth marks. Aardvarks and nine-banded armadillos are similar in that both are myrmecophagous, fossorial, and have relatively low metabolic rates compared to other mammals.

Recently, Straehl *et al*. [39] described long bone microstructure throughout extant Xenarthra. Straehl *et al*. [39] described the long bones of armadillos as having a general three-layer structure composed of inner lamellar and parallel-fibered bone, mid cortical parallel-fibered bone, and an outer lamellar and parallel-fibered bone layer with cyclical growth marks. In the present study, we found thick endosteal layers of lamellar and parallel-fibered bone comprising the innermost cortex in the largest individuals, in agreement with Straehl *et al*. [39]. Similar to the findings of Straehl *et al*. [39], we found an EFS composed of lamellar and parallel-fibered bone, and our largest specimens have cyclical growth marks, both complete and incomplete, within the EFS. However, individuals sampled in our study primarily had a mid-cortex of CCCB, incongruent with Straehl *et al*. [39]. However, Straehl *et al*. [39] noted “Secondary osteons are poorly differentiated and in certain areas irregularly shaped” when describing the mid-cortex. The irregularly shaped secondary osteons noted by Straehl *et al*. [39] may instead be mid-cortical CCCB: infilling of cavities surrounding trabeculae during CCCB formation can lead to osteon-like structures with both irregular borders and distorted shape profiles. If CCCB was misidentified as secondary osteons by Straehl *et al*. [39], that would explain the discrepancy in cortical organizations described between the present study and former study.

It may also be that our differing results from those of Straehl *et al*. [39] are due to the differences in the time taken to achieve skeletal maturity across the wide geographical range of armadillos [14,76–78]. Nine-banded armadillos often reduce activity on cold winter days, and thus lower food availability during winter months could limit growth [14]. Our study included specimens collected from Tennessee, Georgia, and Oklahoma, but the collection locations of specimens used in Straehl *et al*. [39] are unavailable for comparison. Because nine-banded armadillos living in regions with mild winters could be less limited by food availability, studies of growth strategy across the entire range of *D*. *novemcinctus* could reveal differing selective pressures on armadillo growth patterns [79].

Our study also did not account for possible differences in growth rate based on sex. McDonough *et al*. [80] did not find any significant difference in growth between sexes in a study on juvenile nine-banded armadillo growth. However, McDonough [81] did find a slight difference in body mass between genders during a study on a population of nine-banded armadillos in south Texas. We cannot address sexual dimorphism in the current study due to lack of data on the animals’ gender (gender is known only for 3 of our 6 specimens). Gender should be considered in future work on armadillo bone growth to determine if sexually dimorphic signals in the bone microstructure exist.

Despite the aforementioned shortcomings of age classification based on body mass, Loughry and McDonough [14] state, ‘At present, there is no better way to more precisely age armadillos’. The results of our study so far confirm this observation, as hindlimb bone microstructure does not appear useful in the aging of nine-banded armadillos due to the extensiveness of periosteal erosion, endosteal erosion, and CCCB due to cortical drift. However, incorporation of additional ontogenetic stages and other skeletal elements are necessary before the utility of osteohistology in age determination is assessed for armadillos.

Correlating known-age with growth trends in a species facilitates life history reconstruction [82]. Knowledge of a species’ life history is essential to developing accurate conservation and management strategies [83–86]. For example, Marin-Moratalla et al. [85] used osteohistological analysis of long bones in *Addax nasomaculatus*, a critically endangered herbivore, to reconstruct life history traits for use in conservation management. Within Cingulata, seven species are either near-threatened or vulnerable, and five species lack sufficient data for population analysis [9]. Using bone microstructural analysis to evaluate life history parameters can alleviate some of the need for field studies on elusive species. The nine-banded armadillo is representative of a difficult research subject and a good model on which to test osteohistological examination as a means to fill the gaps in our knowledge of their life history. Sampling the nine-banded armadillo along its entire geographic range may illuminate growth responses to varying climatic regimes, which can then be applied to more elusive cingulates for prediction of possible life history changes due to changing climates. As of now, age cannot be accurately determined in the nine-banded armadillo through osteohistological methods. It is possible that incorporation of additional ontogenetic stages could permit identification of patterns in the formation of CCCB, and a scale of relative maturity produced. Similarly, additional ontogenetic sampling of known-age individuals is required to test the utility of epiphyseal fusion as a means to determine relative age in the nine-banded armadillo.

## Conclusions

We examined changes in bone microstructure in tibiae and femora in an ontogenetic series (n = 6) of the nine-banded armadillo (*D*. *novemcinctus)*, with the intent that nine-banded armadillo osteohistology could be used as a comparative tool for reconstructing the life histories of biologically rare, threatened, or extinct cingulates. In ontogenetically younger individuals, femora and tibiae of nine-banded armadillos are generally composed of compacted coarse cancellous bone with regions of primary woven tissue. Periosteal and endosteal resorption and differential deposition occurs extensively as cortical drift takes place in older individuals. Drift and elongation also result in further compaction of cancellous bone previously located in the metaphysis [33]. Two specimens (UTCM 1557 and OMNH 40175) had a complete external fundamental system in both the tibia and femur, indicating cessation of linear growth and achievement of skeletal maturity. However, the presence of the EFS did not appear consistently in larger individuals, indicating individual variation in growth and timing of skeletal maturity. The three largest specimens had either a double-LAG (UTCM 1557 and OMNH 40175) or two distinct LAGs (OMNH 40173) in the outer cortex that did not continue around the entire circumference of the bone.

Unfortunately, the extensiveness of CCCB and periosteal erosion within tibiae and femora in our sample prevents accurate age determination from these elements, similar to findings from Legendre and Botha-Brink [75]. The results of our pilot ontogenetic study suggest future histology examinations on armadillos and likely other cingulates should include earlier juveniles and control for gender, captivity status, season of death, and latitude, in order to determine the effects of each on long bone growth.

## Supporting information

S1 Fig Transverse sections of sampled femora under linearly polarized light. (A) UTCM 802, (B) UTCM 801, (C) OMNH 39188, (D) UTCM 1557, (E) OMNH 40173, and (F) OMNH 40175. All sections are stained with toluidine blue. FL—femur length. Note–some sections were flipped along the horizontal axis to allow for easier comparisons.
(TIF)

S2 Fig Transverse sections of sampled femora under circularly polarized light. (A) UTCM 802, (B) UTCM 801, (C) OMNH 39188, (D) UTCM 1557, (E) OMNH 40173, and (F) OMNH 40175. All sections are stained with toluidine blue. FL—femur length. Note–some sections were flipped along the horizontal axis to allow for easier comparisons.
(TIF)

S3 Fig Transverse sections of sampled tibiae under linearly polarized light. (A) UTCM 802, (B) UTCM 801, (C) OMNH 39188, (D) UTCM 1557, (E) OMNH 40173, (F) OMNH 40175. All sections stained with toluidine blue. TL = tibia length. Note–some sections were flipped along the horizontal axis to allow for easier comparisons.
(TIF)

S4 Fig Transverse sections of sampled tibiae under circularly polarized light. (A) UTCM 802, (B) UTCM 801, (C) OMNH 39188, (D) UTCM 1557, (E) OMNH 40173, (F) OMNH 40175. All sections stained with toluidine blue. TL = tibia length. Note–some sections were flipped along the horizontal axis to allow for easier comparisons.
(TIF)
