# Supplementary figures and images for: Ontogenetic changes in the long bone microstructure in the nine-banded armadillo (Dasypus novemcinctus)
Source: PLoS One. 2019 Apr 25;14(4):e0215655. doi: 10.1371/journal.pone.0215655 (PMC6483220; doi:10.1371/journal.pone.0215655)

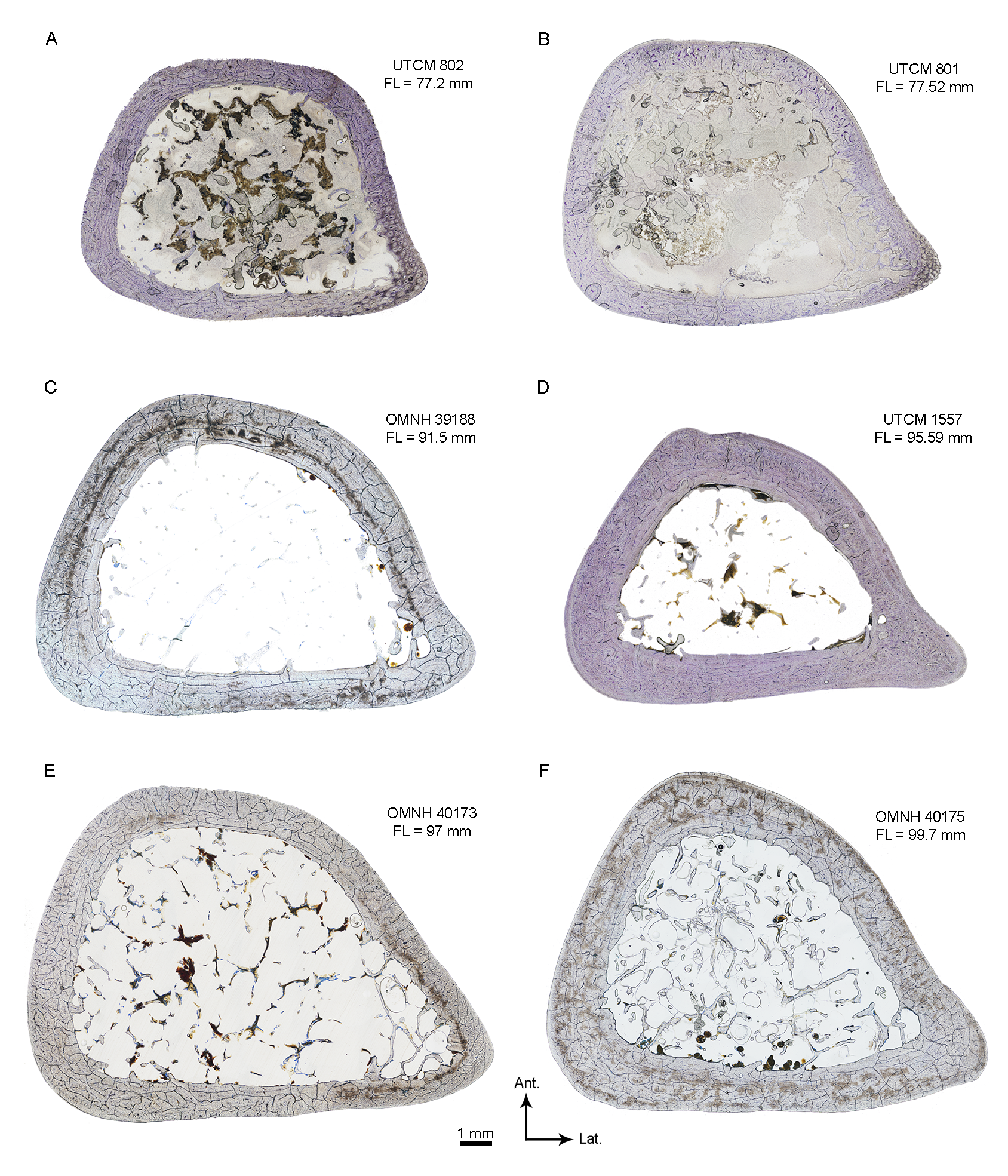

Supplement: S1 Fig — (A) UTCM 802, (B) UTCM 801, (C) OMNH 39188, (D) UTCM 1557, (E) OMNH 40173, and (F) OMNH 40175. All sections are stained with toluidine blue. FL—femur length. Note–some sections were flipped along the horizontal axis to allow for easier comparisons. (TIF) [file pone.0215655.s001.tif]

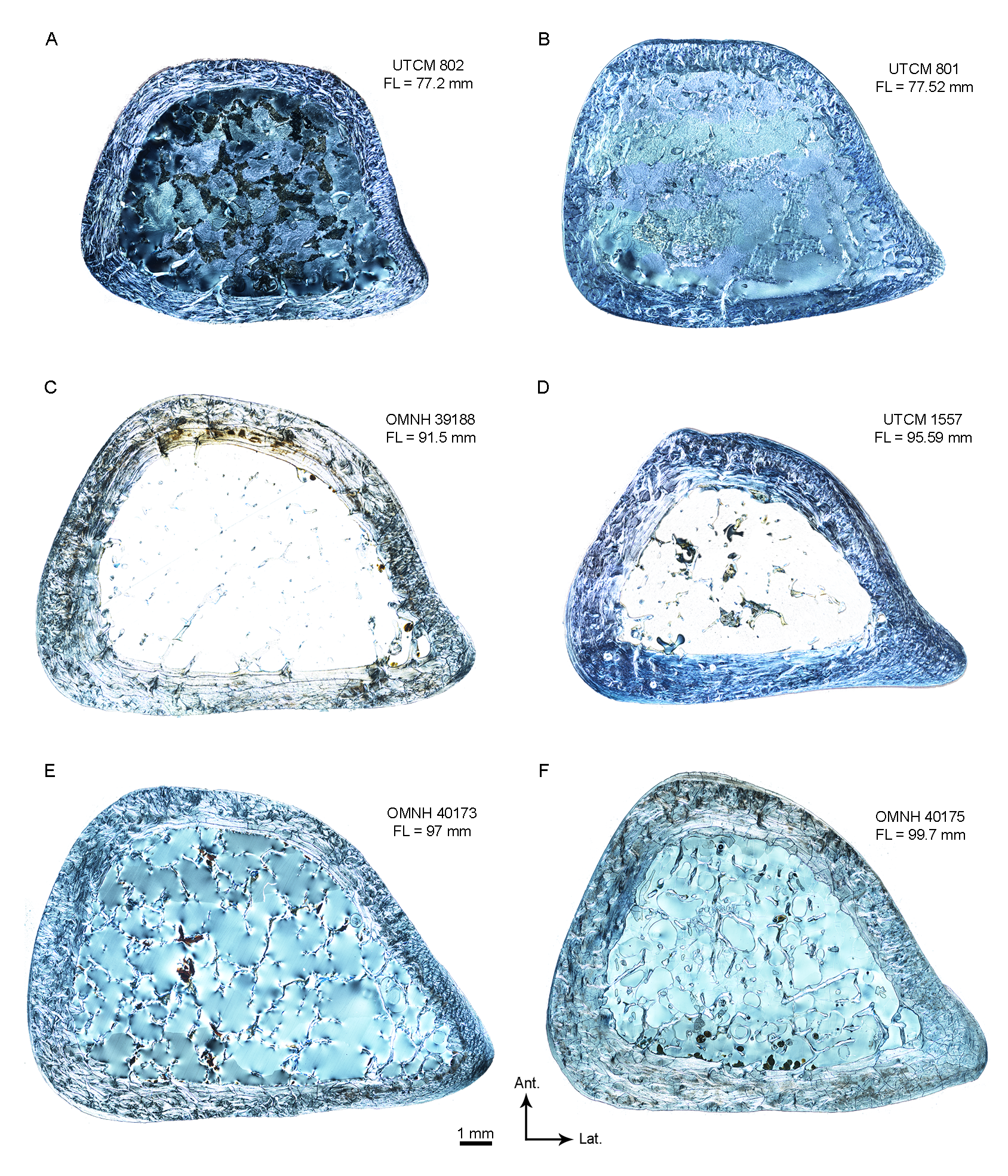

Supplement: S2 Fig — (A) UTCM 802, (B) UTCM 801, (C) OMNH 39188, (D) UTCM 1557, (E) OMNH 40173, and (F) OMNH 40175. All sections are stained with toluidine blue. FL—femur length. Note–some sections were flipped along the horizontal axis to allow for easier comparisons. (TIF) [file pone.0215655.s002.tif]

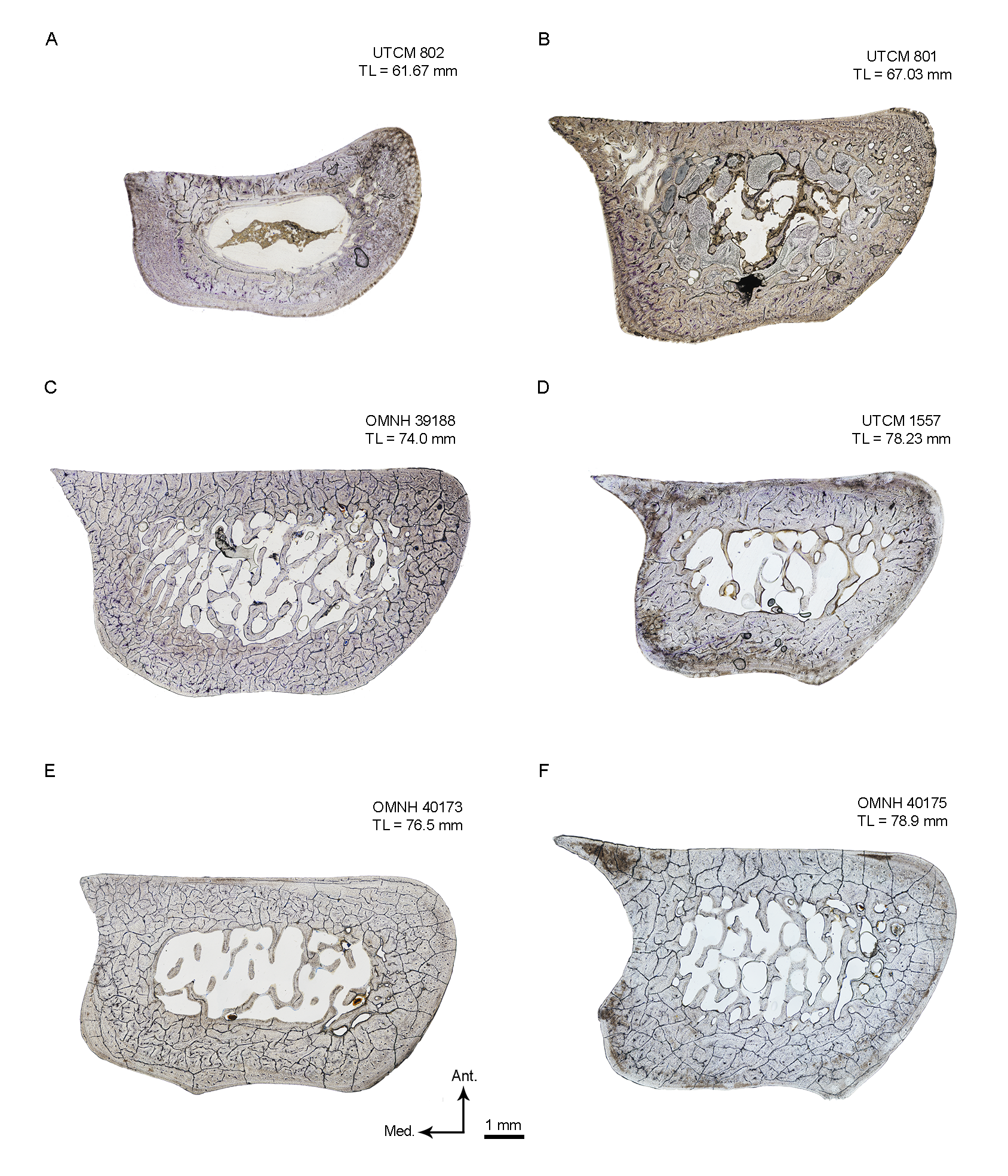

Supplement: S3 Fig — (A) UTCM 802, (B) UTCM 801, (C) OMNH 39188, (D) UTCM 1557, (E) OMNH 40173, (F) OMNH 40175. All sections stained with toluidine blue. TL = tibia length. Note–some sections were flipped along the horizontal axis to allow for easier comparisons. (TIF) [file pone.0215655.s003.tif]

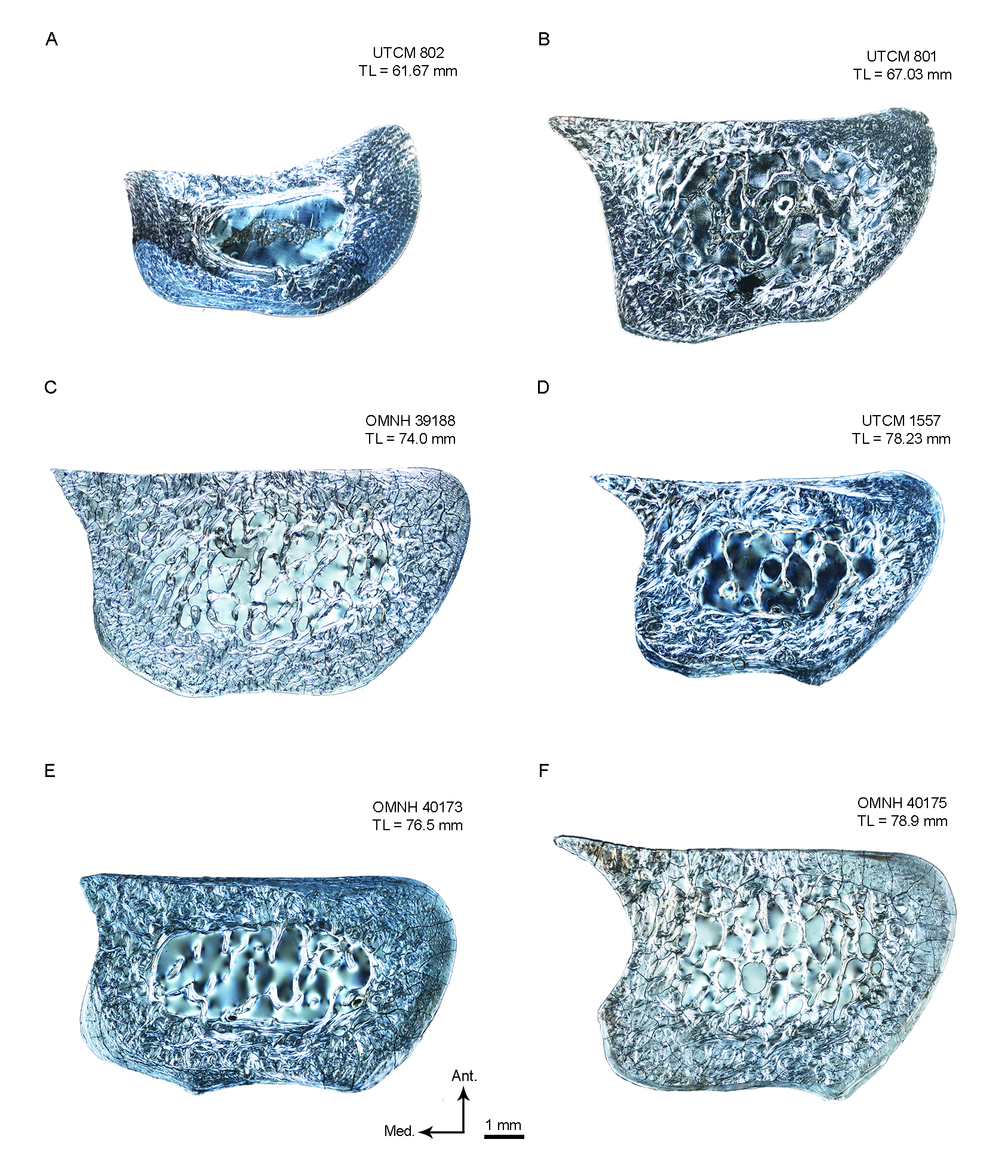

Supplement: S4 Fig — (A) UTCM 802, (B) UTCM 801, (C) OMNH 39188, (D) UTCM 1557, (E) OMNH 40173, (F) OMNH 40175. All sections stained with toluidine blue. TL = tibia length. Note–some sections were flipped along the horizontal axis to allow for easier comparisons. (TIF) [file pone.0215655.s004.tif]
